# Prevalence of Suicidal Ideation in Chinese College Students: A Meta-Analysis

**DOI:** 10.1371/journal.pone.0104368

**Published:** 2014-10-06

**Authors:** Zhan-Zhan Li, Ya-Ming Li, Xian-Yang Lei, Dan Zhang, Li Liu, Si-Yuan Tang, Lizhang Chen

**Affiliations:** 1 Department of Epidemiology and Health Statistics, School of Public Health, Central South University, Changsha, China; 2 Department of Nursing, Central South University, Changsha, Hunan Province, China; 3 The Second Affiliated Hospital, Xiangya Medical School, Central South University, Changsha, Hunan Province, China; University of Vienna, Austria

## Abstract

**Background:**

About 1 million people worldwide commit suicide each year, and college students with suicidal ideation are at high risk of suicide. The prevalence of suicidal ideation in college students has been estimated extensively, but quantitative syntheses of overall prevalence are scarce, especially in China. Accurate estimates of prevalence are important for making public policy. In this paper, we aimed to determine the prevalence of suicidal ideation in Chinese college students.

**Objective and Methods:**

Databases including PubMed, Web of Knowledge, Chinese Web of Knowledge, Wangfang (Chinese database) and Weipu (Chinese database) were systematically reviewed to identify articles published between 2004 to July 2013, in either English or Chinese, reporting prevalence estimates of suicidal ideation among Chinese college students. The strategy also included a secondary search of reference lists of records retrieved from databases. Then the prevalence estimates were summarized using a random effects model. The effects of moderator variables on the prevalence estimates were assessed using a meta-regression model.

**Results:**

**A** total of 41 studies involving 160339 college students were identified, and the prevalence ranged from 1.24% to 26.00%. The overall pooled prevalence of suicidal ideation among Chinese college students was 10.72% (95%CI: 8.41% to 13.28%). We noted substantial heterogeneity in prevalence estimates. Subgroup analyses showed that prevalence of suicidal ideation in females is higher than in males.

**Conclusions:**

The prevalence of suicidal ideation in Chinese college students is relatively high, although the suicide rate is lower compared with the entire society, suggesting the need for local surveys to inform the development of health services for college students.

## Introduction

Suicide is usually not an isolated event, but a sequence of processes starting from death wishes, suicidal ideation, suicidal contemplation, suicide attempt, to suicide completion [1]–[2]. Suicidal ideation, which is defined as thoughts of self-harming or -killing [3] is a significant marker not only for mental-health problems but also for the suicide attempt and completed suicide among youths. Various suicidal cognitions (death wish, suicidal ideation, and suicide plan) and behaviors (suicide attempt, and commit suicide) are determined by common factors, such as personality characteristics, psychopathology, parenting style, family function, and substance [4]–[5]. Thus, it is imperative to identify the characteristics of suicidal ideation and to develop effective prevention and intervention programs.

Currently, suicide is the leading cause of death among 15- to 34-year-old in China, accounting for 19% of deaths in this age group [6]. Suicide has become an important public health issue. A tough challenge placed on top of China's higher education institutions is how to prevent college students from committing suicide and to early detect individuals at high risk of suicide. Given that about 30% of those with suicidal ideation (those who are seriously considering suicide) will attempt suicide, the first step is to determine the overall prevalence of suicidal ideation among college students [7].

Most college students in China are born after post-1980s when the “One-Child” policy switched from a promotive to a mandatory status. Consequently, the majority of them are from single-child families [8]. Hence, it has been becoming a primary argument regarding their relative impulsiveness and inability to withstand negative life events, compared with young adults with siblings. Despite many investigations into the prevalence of suicidal ideation in college students, quantitative syntheses of overall prevalence are scarce, especially in China. Primary prevention as the best and most important strategy requires a sensible plan of action for prevention and improving current policies against suicidal ideation in college students. Therefore, summarizing the prevalence of suicidal ideation in college students is the first step in developing research priorities. We performed a systematic review and meta-analysis of studies on suicidal ideation in Chinese college students with the aim to explore the prevalence of suicidal ideation in this area.

## Materials and Methods

### Literature Search Strategy

Databases including PubMed, Web of Knowledge, Chinese Web of Knowledge, Wangfang (Chinese database) and Weipu (Chinese database) were systematically reviewed to identify articles published between 2004 to July 2013, in either English or Chinese, reporting the estimates of suicidal ideation prevalence in Chinese college students. Articles were identified with search strategy “suicide” OR “suicidal ideation” OR “suicidal tendency” AND (“college student” OR “undergraduate”). The strategy also included a secondary search of reference lists of records retrieved from databases. Two authors screened the titles and abstracts and reviewed the full-text of the eligible articles.

### Inclusion Criteria

The included studies met the following criteria: 1) an original epidemiological study aimed at Chinese college students; 2) with definition of criteria for screening methods and/or screening tools for suicidal ideation and with the provision of description of screening methods; 3) providing information about sample size and prevalence estimation for college students during the prior 12 months; 4) a cross-sectional study or a baseline survey of longitudinal study; 5) a sample size >500. 6) Language is limited to English and Chinese.

### Data Extraction

Information was extracted from all selected publications by two investigators separately. Any disagreement was discussed and resolved by a third investigator. After the duplicates were removed, the following information was extracted from each article: first author, year of publication, region, area (South or North China), age range and mean age if possible, percent of males, major (medical student or not), screening method, screening tool, sample size, response rate, number of people with suicidal ideation, prevalence estimation, and sex-specific prevalence if possible.

### Statistical Analysis

We first transformed proportions into a quantity (the Freeman-Tukey variant of the arcsine square root transformed proportion [9] suitable for the usual fixed and random effects summaries [10]), because the inverse variance weight in fixed-effects meta-analyses is suboptimum when dealing with binary data with low prevalence. Additionally, the transformed prevalence is weighted very slightly towards 50% and thus studies with prevalence of 0 can be included in the analysis. The pooled proportion is calculated as the back-transform of the weighted mean of the transformed proportions, using inverse arcsine variance weights for the fixed effect's model and DerSimonian-Laird weights for the random effect's model.

According to the expected heterogeneity across studies, a random-effects model was used to calculate pooled prevalence and 95% confidence interval (CIs). Between-study heterogeneity was evaluated with the Cochran chi-square (χ^2^) and quantified with the *I^2^* statistic, which will be used to estimate total variation across studies due to heterogeneity rather than chance (<25% is considered as low heterogeneity, 25%–50% as moderate, and >50% as high) [11], [12]. In order to understand the prevalence of suicidal ideation in different sex, mean age, study year, sample, area and major and also explore the potential heterogeneity between studies, dummy-coding is used for three or more categories variables. We conducted subgroups analysis. Then the effects of these covariates on the logit prevalence of the outcome were estimated using meta-regression. Each covariate was included separately in univariate analyses, and then a multivariable meta-regression model was constructed including all covariates. Publication bias was evaluated Begg's test, the modified Egger's linear regression test and Trim-and-fill approach. Significance was set at *P*<0.05. All statistical calculations were made using Stata 11.0 (College Station, Texas) and Statsdirect 2.7.9 (http://www.statsdirect.com).

## Results

### Study flow and characteristics

**Figure 1:**
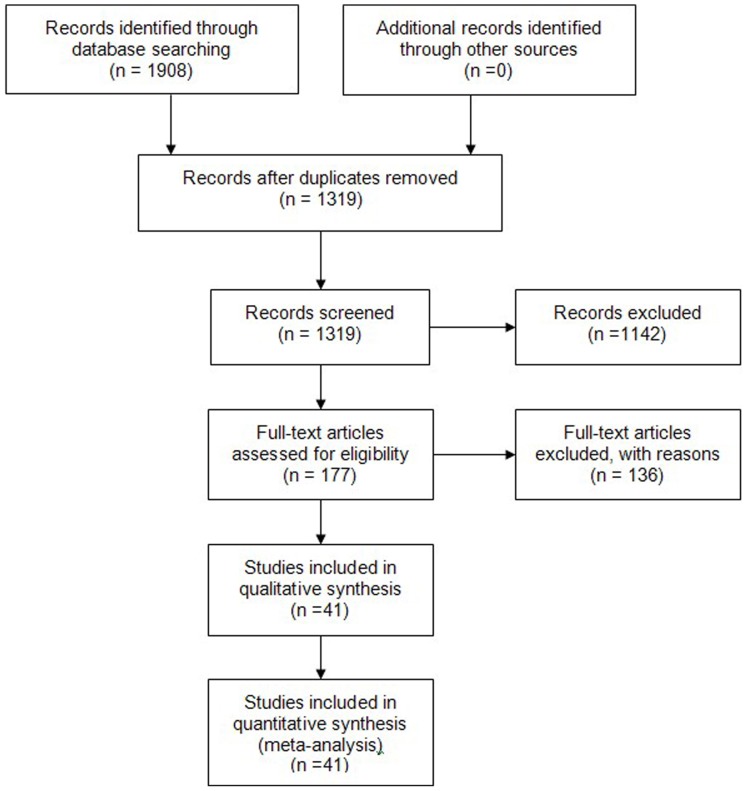
Flow diagram of included/excluded studies.

A total of 1908 studies were identified after initial search. After removal of duplicates and an initial screening, we reviewed 177 papers in full. After exclusion of ineligible reports, 41 studies [13]–[53] published between January 2004 and July 20, 2013 were finally included. The flow diagram of the search process is exhibited in Figure 1. The characteristics of studies on the prevalence of suicidal ideation among college students are shown in Table 1. The sample size of the reviewed studies ranged from 610 to 21072 (median 2374), with a total of 160339 college students. Among them, 28 (68.3%) reports reported data on male (*n* = 69701), 28 (68.3%) reports on female (*n* = 55140), and 13 (31.7%) reports included mixed sex population size (*n* = 35228). In the surveys with samples, more than 54% of the individuals were men. The age ranged from 16 to 25 years old. Among them, 29 (70.7%) reports were aimed at South China (*n* = 112706), 12 reports at North China (*n* = 47633), 16 (39.0%) at medical universities (*n* = 51645), and 25 (61.0%) at non-medical students (*n* = 108694). Table 1 shows details from the 41 selected studies. Questionnaire is used to collect the information in the most of studies. 18 use the self-made scale, 9 for beck scale for suicide ideation-Chinese version, 7 for university personality inventory, and the left use other scales.

**Table 1:** Characteristic of Studies on the Prevalence of Suicidal Ideation among College Students.

NO.	First author	Publication year	Region	Province	Area	Age (mean years)	Male Percent (%)	Medical student	Screening method	Screening tools	Response Rate (%)	Suicidal ideation	Sample size	Prevalence (%)
1	Wang [13]	2012	Mainland	Henan	Northern	17–22	44.2	No	Q	UPI	97.10	162	3850	4.21
2	Chen [14]	2010	Mainland	Chongqing	Southern	20.0	56.6	Yes	Q	SMQ	81.0	1279	9808	13.0
3	Xu [15]	2004	Mainland	Hunan	Southern	-	53.4	Yes	Q	SMQ	97.9	89	610	14.6
4	Chen [16]	2008	Mainland	Jilin	Northern	21.3	67.1	Yes	QI	CIDI	98.9	97	1822	5.3
5	Liu [17]	2007	Mainland	Yunnan	Southern	21.2	50.4	No	Q	SMQ	97.4	437	3313	13.2
6	Chao [18]	2008	Mainland	Jiangxi	Southern	-	51.8	No	Q	SMQ	84.2	113	1010	11.2
7	Shang [19]	2008	Mainland	Gansu	Northern	20.5	35.8	Yes	Q	SMQ	92.3	411	2678	15.4
8	Qian [20]	2008	Mainland	Beijing	Northern	-	35.4	Yes	Q	SMQ	88.2	122	2199	5.6
9	Yang [21]	2013	Mainland	Henan	Southern	20.7	33.5	Yes	Q	UPI	92.2	110	1372	8.0
10	Zhang [22]	2007	Mainland	Guangdong	Southern	-	52.8	Yes	Q	EPQ	99.3	1763	7189	24.5
11	Wang [23]	2011	Mainland	Yunnan	Southern	18.9	35.5	Yes	Q	SMQ	99.84	409	1850	22.1
12	Jiang [24]	2006	Mainland	Fujian	Southern	-	56.3	Yes	Q	SMQ	90.4	171	1254	13.6
13	Li [25]	2009	Mainland	Beijing	Northern	25.1	64.4	No	Q	UPI	97.6	380	21072	1.8
14	Li [26]	2007	Mainland	Zhejiang	Southern	18.6	40.1	No	Q	SCL-90	95.0	800	8160	9.8
15	Bao [27]	2009	Mainland	Anhui	Southern	20.1	61.0	Yes	Q	BSSI	92.7	217	1529	14.2
16	Huang [28]	2007	Mainland	Zhejiang	Southern	18.85	64.0	No	QI	SCL-90	94.2	219	3564	6.1
17	Wang [29]	2011	Mainland	Shanxi	Northern	19.2	44.9	Yes	Q	SDSS	98.3	74	1177	6.3
18	Kong [30]	2012	Mainland	Anhui	Southern	20.4	42.9	Yes	Q	SMQ	91.8	444	3030	14.7
19	Song [31]	2010	Mainland	Anhui	Southern	21.8	55.9	Yes	Q	BSSI	95.9	210	2062	10.2
20	Sun [32]	2010	Mainland	Shandong	Northern	20.4	42.4	No	Q	BSSI	94.3	228	1886	12.1
21	Yang [33]	2010	Mainland	Harbin	Northern	21.3	51.1	No	Q	SMQ	87.8	479	5240	9.1
22	Yan [34]	2009	Mainland	Guangdong	Southern	21.2	46.8	No	Q	SMQ	87.2	57	772	7.4
23	Shi [35]	2007	Mainland	Guangdong	Southern	20.6	49.8	No	Q	SMQ	98.0	297	2564	11.6
24	Shi [36]	2013	Mainland	Guangxi	Southern	20.4	52.7	No	Q	BSSI	91.0	434	2730	15.9
25	Ran [37]	2006	Mainland	Sichuan	Southern	-	70.6	No	Q	UPI	100.0	229	8850	2.6
26	Kan [38]	2013	Mainland	Zhejiang	Southern	-	46.1	No	Q	UPI	99.9	263	10138	2.6
27	Zhao [39]	2011	Mainland	Guangdong	Southern	21.7	56.6	No	Q	SMQ	96.35	98	1450	6.8
28	Yin [40]	2009	Mainland	Jiangsu	Southern	21.3	36.4	No	QI	SMQ	95.7	90	670	13.4
29	Yang [41]	2007	Mainland	Henan	Northern	16–24	74.6	No	Q	SCL-90	93.9	705	3568	19.8
30	Liu [42]	2013	Mainland	Guangdong	Southern	14–25	58.5	No	Q	SPS	100.0	585	6154	9.5
31	Tu [43]	2013	Mainland	Guangdong	Southern	21.0	60.0	No	Q	UPI	100.0	144	11603	1.24
32	Liu [44]	2008	Mainland	Beijing	Northern	-	42.5	Yes	Q	SMQ	91.4	125	1204	10.4
33	Li [45]	2008	Mainland	Beijing	Northern	20.0	55.6	No	Q	BSSI	97.6	166	2055	8.1
34	Cao [46]	2009	Mainland	Anhui	Southern	19.3	46.2	Yes	QI	BSSI	91.8	1669	10344	16.4
35	Fan [47]	2008	Mainland	Anhui	Southern	20.3	53.5	Yes	Q	BSSI	94.4	496	3517	14.1
36	Tang [48]	2011	Mainland	Hubei	Southern	10–24	54.7	No	Q	SMQ	100.0	178	2013	8.8
37	Zhang [49]	2012	Mainland	Guangdong	Southern	21.8	48.6	No	Q	SMQ	87.2	137	689	19.9
38	Zhu [50]	2011	Mainland	Jiangsu	Southern	19.6	47.1	No	Q	BSSI	98.1	430	2374	18.1
39	Zhao [51]	2012	Mainland	Guangdong	Southern	19.5	46.4	No	Q	BSSI	84.0	225	1168	19.3
40	Wang [52]	2010	Mainland	Shanxi	Northern	19.0	46.9	No	Q	UPI	93.0	46	882	5.6
41	Gau [53]	2008	Taiwan	Taiwan	Southern	19.4	48.5	No	Q	SMQ	79.2	759	2919	26.0

Screen methods: Q, questionnaire distribution; QI, questionnaire-based interview. Screening tools: UPI, University Personality Inventory, SMQ, Self-made scale; CIDI, Composite International Diagnostic Interview; BSSI, Beck Scale for Suicide Ideation-Chinese Version.

### Pooled analysis in different category

#### Overall prevalence

**Figure 2:**
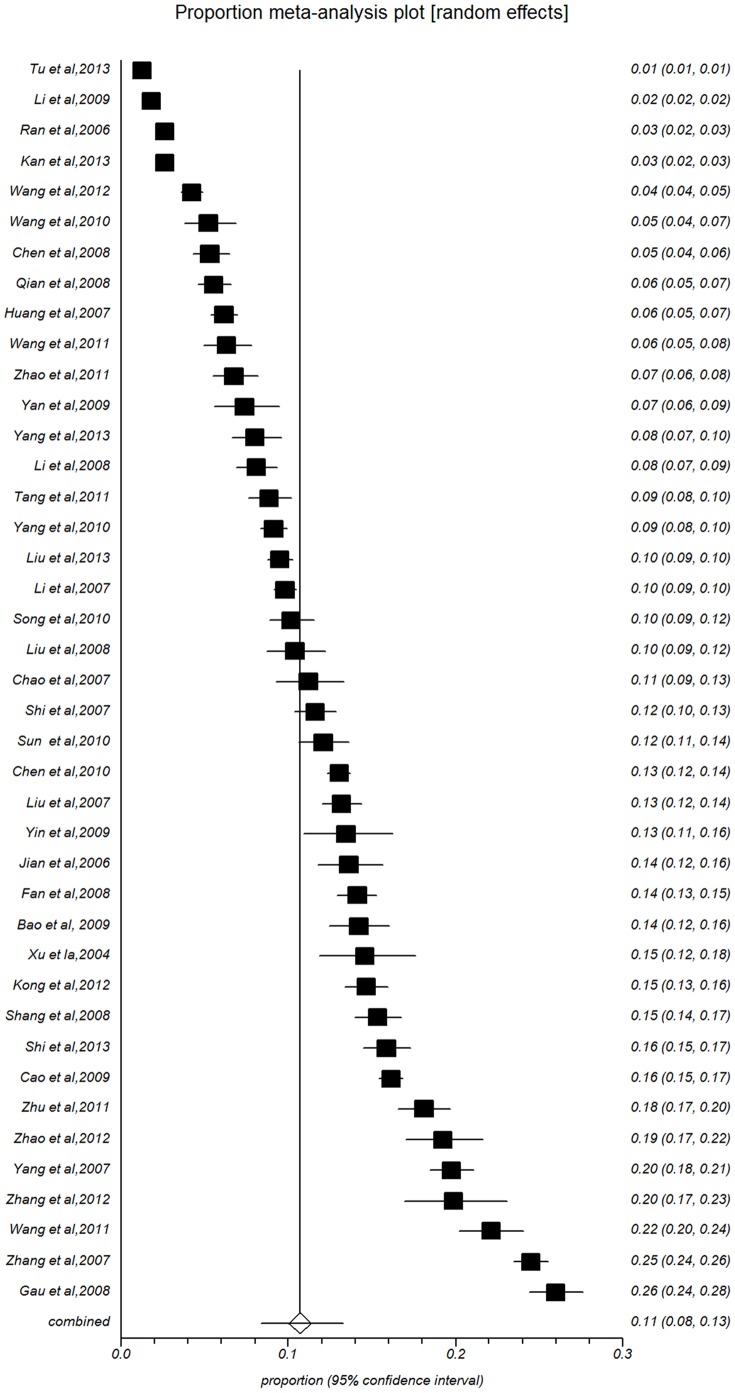
Forest plot of prevalence of suicidal ideation for total people.

The point prevalence of suicidal ideation with the 41 individual study populations ranged between 1.24% and 26.00%, with an overall meta-analysis prevalence of 10.72% (95%CI: 8.41%–13.28%, Figure 2) and evident high-level heterogeneity between studies (*I^2^* = 99.6%, *P*<0.0001).

#### Sex

**Figure 3:**
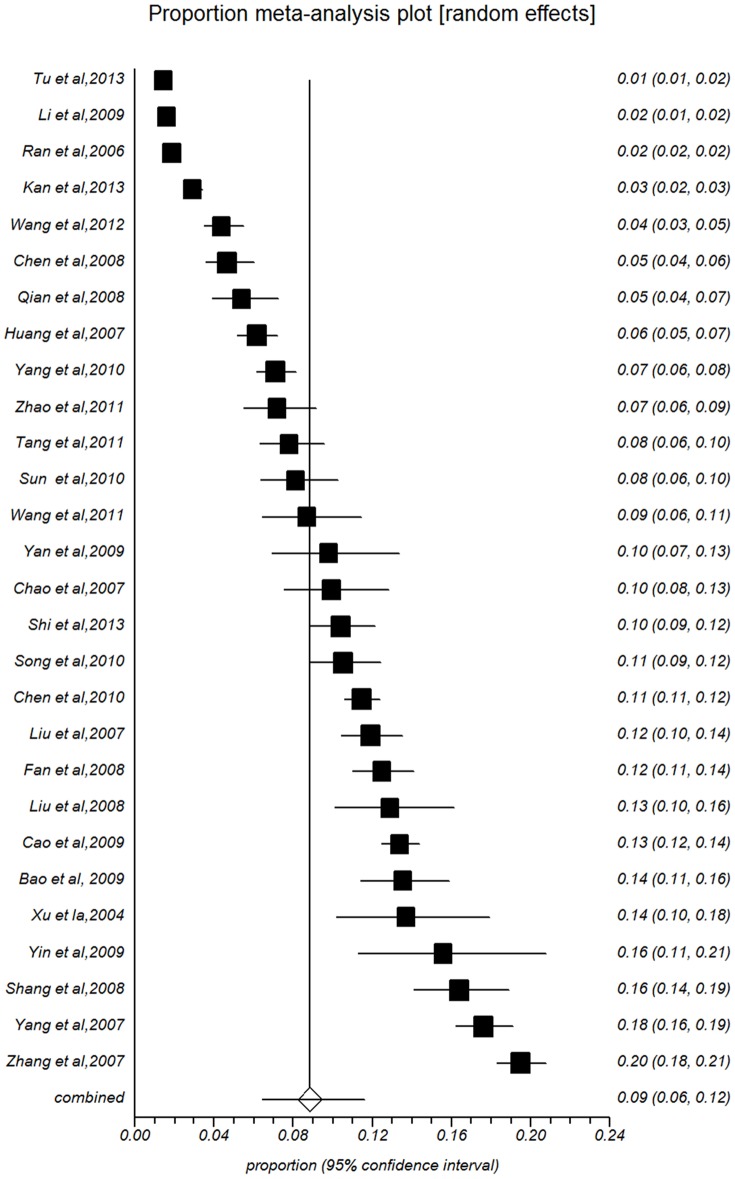
Forest plot of prevalence of suicidal ideation for male.

**Figure 4:**
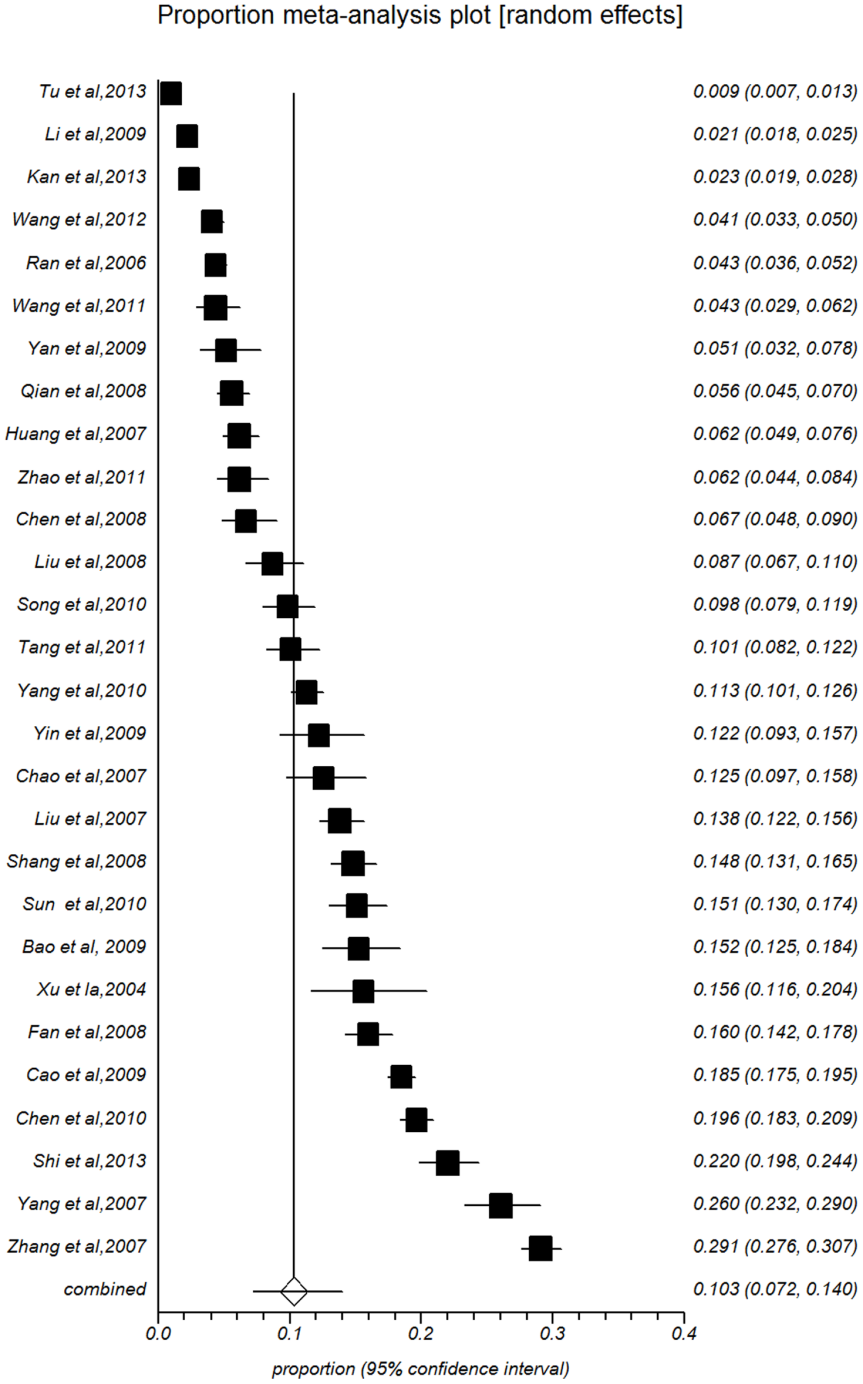
Forest plot of prevalence of suicidal ideation for female.

Pooled prevalence of all subgroups of study year, population size, male percent, area, major and sex are presented in Table 2. The summarized prevalence of females (10.32%, 95%CI: 7.17%–13.97%, Figure 3) was higher than that of males (8.84%, 95%CI: 6.45%–11.57%, Figure 4). The prevalence estimates for studies with more than 50% males were lower than estimates of groups with less than 50% males (8.84%, 95%CI: 6.45%–11.57%).

**Table 2:** Prevalence of Suicidal Ideation among College Students According to Different Category.

Category	Subgroup	NO. of Studies	Prevalence (*95%CI*)(%)	*N*	*I^2^* (%)	*P*	Publication Bias Test	
							*P*(Begg's Test)	*P*(Egger's Test)
	Total	41	10.72[8.41–13.28]	160339	99.6	<0.0001	0.258	0.008
Study year	2004–2007	9	12.10[6.97–18.44]	39072	99.7	<0.0001	0.915	0.704
	2008–2009	13	10.73[6.16–16.39]	51791	99.7	<0.0001	0.858	0.183
	2010–2011	10	10.77[8.30–13.53]	28742	97.9	<0.0001	0.600	0.942
	2012–2013	9	9.35[5.13–14.68]	47034	99.6	<0.0001	0.119	0.009
Sample size	<2000	16	11.40[8.89–14.19]	19345	97.5	<0.0001	0.003	0.001
	2000–4000	15	12.22[9.30–15.49]	42436	99.0	<0.0001	0.0001	0.0001
	>4000	10	7.70[3.77–12.85]	98558	99.9	<0.0001	0.009	0.0001
Male percent	<50%	20	11.67[8.63–15.10]	59926	99.3	<0.0001	0.186	0.388
	≥50%	21	9.85[6.68–13.57]	100413	99.7	<0.0001	0.492	0.069
Area	Northern	12	8.00[4.67–12.14]	47633	99.5	<0.0001	0.381	0.065
	Southern	29	11.95[9.09–15.14]	112706	99.6	<0.0001	0.838	0.119
Medical students	Yes	16	12.58[10.09–15.30]	51645	98.7	<0.0001	0.757	0.792
	No	25	9.61[6.97–12.62]	108694	99.6	<0.0001	0.083	0.008
Sex	Male	28	8.84 [6.45–11.57]	69701	99.3	<0.0001	0.570	0.0001
	Female	28	10.32[7.17–13.97]	55140	99.4	<0.0001	0.101	0.422
Response rate	90%≤	9	12.81[9.30–17.32]	25059	98.7	<0.0001	0.531	0.589
	<90%	32	10.93[9.72–12.13]	135280	99.5	<0.0001	0.023	0.004

#### Periods

The pooled prevalence estimate decreased over time. Between 2004 and 2007, the pooled prevalence estimate was 12.10% (*95%CI:* 6.97%–18.44%), which decreased to 10.73% (95%CI: 6.16%–16.39%) between 2008 and 2009. The estimate was 10.77% (95%CI: 8.30%–13.53%) in 2010 to 2011, and 9.35% (95%CI: 5.13%–14.68%) in 2012 to 2013.

#### Major and area

The prevalence for the non-medical group (9.61%, 95%CI: 6.97%–12.62%) was smaller than medical group (12.58 95%CI: 10.09%–15.30%). Prevalence among college students from South and North China were 11.95% (95%CI: 9.09%–15.14%) and 8.00% (95%CI: 4.67%–12.14%) respectively.

#### Population size and response rate

Regarding population size, the pooled prevalence was 11.40% (95%CI: 8.89%–14.19%) for population size <2000, 12.22% (95%CI: 9.30%–15.49%) and 7.70% (95%CI: 3.77%–12.85%) for population size >4000. Table 2 shows information about heterogeneity and publication bias. The pooled prevalence for response rate ≤90% was 12.81% (95%CI: 9.30–17.32) and 10.93 (95%CI: 9.72–12.13) for response rate >90%.

### Meta-regression analysis, assessment of publication bias

**Figure 5:**
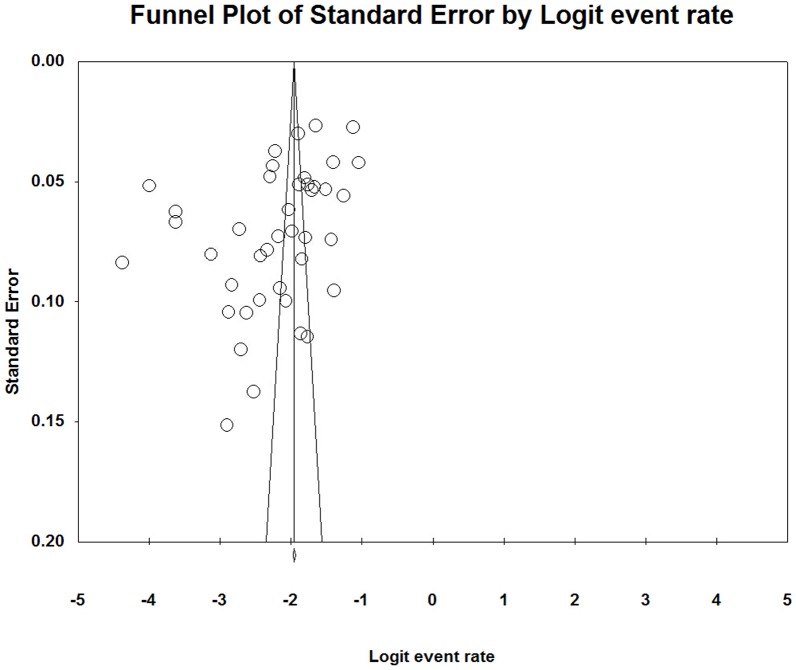
Funnel plot of studies included in the synthesis.

We noted significant heterogeneity within studies and subgroups (*P*<0.001, *I^2^* = 97.9%–99.0%). In the univariate meta-regression analyses (Table 3), year of publication, male percent and major used to define suicidal ideation did not modify the estimate of prevalence. In three population size groups (<2000, 2000–4000, >4000), the prevalence estimates in population size >4000 were significantly lower compared with the other two groups. Mean age (*P* = 0.017) and response rates (*P* = 0.012) were significantly associated with the prevalence estimates. A meta-regression model was constructed including male ratio, response rate and population size (2000–4000). This model explained much of the heterogeneity between studies (*R^2^* = 52.85%, *P* = 0.016, Table 3). We noted the prevalence of suicidal ideation decrease with response rate and male rate increase in the proportion and increase with the sample size increase. Then bias of publication was assessed using Begg's funnel plot and Egger's test. Significant publication bias was indicated by Begg's test (*P* = 0.008), but not by the modified Egger's linear regression test (*P* = 0.258). The funnel plot an apparent asymmetry that suggested the presence of a potential publication bias (Figure 5). Trim-and-Fill analyses was also used to assess publication bias. The pooled estimations for fixed and random models were −2.10 (95%CI: −2.17 to −2.02) and −2.21 (95%CI: −2.45 to −1.97). After estimated missing study was included into the meta-analysis, the pooled estimations for fixed and random models were −2.40 (95%CI: −2.47 to −2.33) and −2.43 (95%CI: −2.68 to −2.17). The estimated missing number of study is eight, which suggested the presence of publication bias.

**Table 3:** Results of Meta-regression for Prevalence among Chinese College Students.

Covariate	Meta-regression coefficient	*95%CI*	*P* value	Variance explained (%)
**Univariate analyses**				
Year of publication				
2004–2007	0.176	−0.356 to 0.709	0.507	−1.40
2008–2009	0.015	−0.460 to 0.492	0.946	−2.55
2010–2011	0.078	−0.437 to 0.594	0.759	−2.31
2012–2013	−0.281	−0.809 to 0.246	0.288	0.41
Male percent	−0.235	−0.672 to 0.202	0.283	0.46
Sample size				
<2000	0.192	−0.258 to 0.642	0.394	−0.64
2000–4000	0.283	−0.168 to 0.734	0.212	1.51
>4000	−0.604	−1.082 to −0.126	0.015	12.16
Area	0.392	−0.077 to 0.863	0.099	4.43
Mean age	−0.230	−0.417 to −0.044	0.017	16.2
**Screening tool**	−0.435	−0.859 to −0.010	0.045	7.26
Response rate	−0.050	−0.088 to −0.011	0.012	13.01
Medical student	0.359	−0.080 to 0.799	0.106	4.16
**Multivariable analyses**			0.016	52.85
Response rate	−0.057	−0.10 to −0.014	0.011	
Male rate	−0.030	−0.055 to −0.004	0.024	
Sample size(2000–4000)	0.778	0.210 to 1.346	0.010	

## Discussion

There is a lack of nationwide data regarding prevalence of suicidal ideation in Chinese college students at present. This is the first report attempting to synthesize the prevalence estimations of suicidal ideation among Chinese college students using meta-analysis. This comprehensive systematic review with meta-analysis of observational studies done in China in the last decade included 41 reports and more than 160 thousand college students. Therefore, it was possible to provide a reliable estimate of prevalence. This meta-analysis indicates that the prevalence of suicidal ideation in Chinese college students is 10.72% (95%CI: 8.41%–13.28%), which was close to previously reported rates [31] and fell in the range of 6%–39.2% reported in other countries [54]–[56].

Suicidal ideation among college students is associated with various factors, which can be categorized into several domains, including physiology, suicide attitude, psychological health status, stress, and social support.

Sex may significantly affect the prevalence of suicidal ideation. A national representative population-based study from Korea found the prevalence of suicidal ideation was significantly higher among females: about 19% of females and 11% of males reported suicidal ideation [57]. In Kampala (Uganda), the prevalence of suicidal ideation was significantly higher in girls (34%) than boys (23.2%) [58]. However, other studies did not report the sex difference for suicidal ideation [59]. This meta-analysis showed that 8.84% of males and 10.32% of females had suicidal ideation, and this difference can be partially explained by the following reasons. According to the gender role socialization theory, males are expected to be independent and decisive, and display masculinity (e.g. engagement in risky behaviors). In contrast, females are expected to be dependent and indecisive, and express their stress via rumination. So females have a higher rate of suicide attempts than males, while males have a higher rate of mortality from suicide than females [60]. In China, fatal suicide rate for male was higher than females. But fatal suicide rate for females was still higher than males among population aged 15 to 34 (including college student) [61]. Suicidal ideation pattern in this meta-analysis may be similar to the pattern of fatal suicide.

Psychological health status exerts an implicit, deep and fundamental influence on suicidal ideation. About 50%–75% of children and adolescents who have suicidal ideation suffered from emotional disturbance, especially severe depression; about 1/4–2/3 of adolescents with suicidal ideation have a history of drug dependence and abuse [62]. Personality disorder in adults with suicidal ideation is more common than adolescents, indicating that adolescents with personality disorder are easily addicted to drug abuse and more likely to develop suicidal ideation [63]. Sexual orientation will greatly affect the suicidal ideation and suicidal behaviors of the youths, which is regulated by depression, hopelessness and drug abuse [64]–[65].

Negative life events that are a direct source of stimulation inducing suicidal ideation. College students who experience more negative life events are at increased risk for suicidal ideation. Associations between suicidal ideation and failure in love, financial problems, and divorce/separation of parents have been reported [71]. Findings also suggest that negative life events may be associated with suicidal ideation among adolescent, depressive symptoms were associated with suicidality and depressive symptoms may mediate the association of minor negative life events with suicidality [66].

Higher levels of family cohesion and family support were associated with lower levels of suicidal ideation and depression [67]. Living alone increased the risk for suicidal ideation among college students [68], suggesting that facilitating access to support resources might reduce the risk for suicidal behavior among college students. In addition, friends are also an important source of support and may be an even greater resource than the family [69]. Therefore, evaluating the support system of friends may provide further information that can help prevent suicide.

There are other factors of suicidal ideation such as cultural orientation [70], religious belief [51], history of family suicide [36], and social-economic status [46]. The factors affecting suicidal ideation in college students are not single, but multifaceted. Depression fully mediated the relationship between physical illness, interpersonal conflict and suicidal ideation, but not the relationship between financial problem and suicidal ideation [71]. Another study confirmed the hypothesis that family cohesion and social self-concept were significant moderators for children and adolescents [70]. In sum, on an individual basis, one or all three factors could work, and even accidental factors may also contribute to suicidal ideation.

For the reasons given above, some effective measures should be taken. It is suggested that the experience of violence had a significant negative direct effect and peer support had a significant positive direct effect on their happiness. Happiness had a significant negative effect and the experience of violence had a significant positive effect on suicidal ideation. These findings demonstrate the fundamental importance of reducing exposure of violence to the youth, and that increasing peer support and their happiness may be the key to adolescent suicidal ideation prevention [72]. Sakamoto et al found that watching the video had substantial psych educational effects, which could be considered as prevention measures [73]. What is more, the lack of mental health services is an urgent problem of campus populations in China. More mental health counselors are needed [74]. College students would benefit greatly by attention to this measures.

Although this meta-analysis includes 41 studies encompassing a larger sample size than individual studies, there are still some limitations. First, the heterogeneity of both total population and subgroup was high. Most of the included studies had large sample sizes that produced very precise estimates. Meta-regression analysis showed that male ratio, sample size and response rate may be associated with the prevalence of suicidal ideation, which explained about the half of the heterogeneity between studies. But non-theory-guided selection of predictors based on an algorithm may lead to replication difficulties in future studies. We noted the prevalence of suicidal ideation decrease with response rate and male rate increase in the proportion and increase with the sample size increase. The difference of screening methods may have an influence on the heterogeneity. But this result should be carefully explained. Because some variable are associated with outcome variables in a single, and not associated with outcome variable after being combined. We call this aggregation bias, ecological bias, ecological confounding or ecological fallacy. It is acknowledged that there must be some factors affecting the heterogeneity. According to the present data, we cannot further identify the source of heterogeneity. Second, results from Begg's funnel plot and Egger's test are different but the funnel plot and Trim and Fill methods suggested the presence of a potential publication bias, a language bias, and inflated estimates by a flawed methodological design in smaller studies. The available studies had important methodological limitations, particularly related to college students with suicidal ideation selection and recruitment, which may have led to subjects included in studies differing in important ways from those excluded or ineligible for analyses, and that may have unpredictably affected prevalence estimates. Finally, the studies with a sample size less than 500 subjects were not included on the basis of the sake of representativeness of survey samples. The information bias may still affect the pooled results, although we restricted sampling methods in inclusion criteria.

In conclusion, the prevalence of suicidal ideation in Chinese college students is relatively high, although the suicide rate is lower than that of the entire society. Considering the policy of enrollment expansion of higher education in China is implemented, It is sincerely hoped that this meta-analysis synthesized within the past decade will serve as a “wake-up” call to university administration alike. The results should encourage not only more needed research in terms of the college student group as a distinct population unto itself, but also prompt colleges, society and family to develop and implement programs to address, educate and prevent those college students who are most at risk for suicidal ideation.

## Supporting Information

Checklist S1 PRISMA 2009 Checklist.
(DOC)

Diagram S1 PRISMA 2009 Flow Diagram.
(DOC)
