# Supplementary material for: Prevalence of Suicidal Ideation in Chinese College Students: A Meta-Analysis
Source: PLoS One. 2014 Oct 6;9(10):e104368. doi: 10.1371/journal.pone.0104368 (PMC4186746; doi:10.1371/journal.pone.0104368)
Supplement: Diagram S1 — PRISMA 2009 Flow Diagram. (DOC) [file pone.0104368.s002.doc]

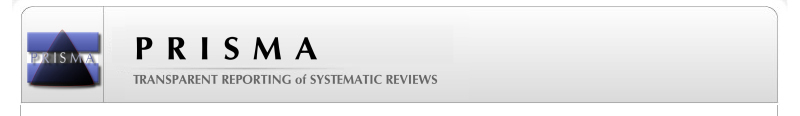
**PRISMA 2009 Flow Diagram**

**Screening**

**Included**

**Eligibility**

**Identification**

Records identified through database searching
(n = 1908)

Additional records identified through other sources
(n =0)

Records after duplicates removed
(n = 1319)

Records screened
(n = 1319)

Records excluded
(n =1142)

Full-text articles assessed for eligibility
(n = 177)

Full-text articles excluded, with reasons
(n = 136)

Studies included in qualitative synthesis
(n =41)

Studies included in quantitative synthesis (meta-analysis)
(n =41)
